# Long-term activation of the pro-coagulant response after neoadjuvant chemoradiation and major cancer surgery

**DOI:** 10.1038/sj.bjc.6605463

**Published:** 2009-12-01

**Authors:** M Byrne, J V Reynolds, J S O'Donnell, M Keogan, B White, M Byrne, S Murphy, S G Maher, G P Pidgeon

**Affiliations:** 1 Department of Surgery, St James's Hospital, Trinity College, Dublin, Ireland; 2 Department of Haematology, St James's Hospital, Trinity College, Dublin, Ireland

**Keywords:** oesophageal cancer, multimodal, pro-coagulant, immunoinflammation

## Abstract

**Background:**

The association between cancer, major surgery and venous thromboembolism (VTE) is well established. Multimodal therapy is increasingly being used as standard treatment for localised gastrointestinal cancer. The aim of this study was to examine the markers of pro-coagulation response and VTE risk in an exemplar multimodal model of pre-operative combination chemotherapy and radiation therapy, followed by complex cancer surgery.

**Methods:**

Consecutive patients (*n*=36) with localised oesophageal cancer were studied at baseline after the first and second cycles of chemoradiation, and on post-operative days 1–28, and at 3, 6 and 9 months. Factors regulating the pro- and anti-coagulant response, as well as pro-inflammatory markers including NF*κ*B activation in peripheral blood mononuclear cells, were examined. All patients received enoxaparin 40 mg s.c. postoperatively up to discharge, and underwent pulmonary CT-pulmonary angiography and venography on day 10 postoperatively.

**Results:**

Four (11%) non-fatal thromboembolic events were documented, all after hospital discharge. Neoadjuvant therapy before surgery activated factor VIII (FVIII) and pro-inflammatory NF*κ*B, and increased D-dimers, pro-thrombin fragment 1+2 (F1+2) and the thrombin-anti-thrombin complex (TAT). Surgery significantly (*P*<0.05) increased pro-thrombin time (PT), activated partial thromboplastin time, fibrinogen, D-dimers, TAT, F1+2 and FVIII up to 6 months.

**Conclusion:**

These data highlight the linked pro-coagulant and immunoinflammatory pathways in the multimodal management of oesophageal cancer, and suggest that the duration of current standard thromboprophylaxis regimens warrants further study.

The association between cancer and venous thromboembolism (VTE) was established a long time ago (Behanwala and Williamson, 2009). Trousseau reported the association between phlegmasia alba dolens and advanced malignancy in 1865, and James and Matheson reported the association of VTE with occult malignancy in 1935 (Trousseau, 1865; Illtyd James and Matheson, 1935). The odds ratio for VTE in malignant disease is approximately 6.5, and VTE is the second most common cause of death in cancer patients (Heit *et al*, 1999). Cancer is associated with a pro-coagulant response, particularly through tumour elaboration of tissue factor, a tumour- and host immune-mediated pro-inflammatory cytokine response and platelet activation. This may manifest in measures of blood-clotting activation, including pro-thrombin factors 1 and 2 (F1 and 2), Factor VIII (FVIII:C) and the thrombin-anti-thrombin (TAT) complex (Hoffman *et al*, 2001; Rickles and Falanga, 2001; Zwicker and Furie, 2007). All cancer treatment modalities such as surgery, chemotherapy and radiation therapy can induce a pro-coagulant and pro-inflammatory response (Geerts *et al*, 2001).

Cancer surgery is associated with an approximate doubling of the VTE risk compared with non-cancer surgery (Rickles and Levine, 2001). The triad of factors associated with coagulation postulated by Virchow, including venous stasis, activation of coagulation and inflammatory pathways, as well as an associated shift to a pro-coagulant endothelium, are all relevant to cancer surgery. In a multivariate analysis, five risk factors were identified and associated with VTE risk in surgical cancer patients: age above 60 years, previous VTE, advanced disease, anaesthesia administered longer than 2 h and bed rest longer than 3 days (Agnelli *et al*, 2006). Previous studies carried out up to 4 weeks postoperatively showed an ongoing activation of coagulation (Galster *et al*, 2000). New insights highlight the interaction between the inflammatory and coagulant response, particularly after surgery, and this association is underlined by randomised trials that show that recombinant activated protein C (APC) significantly reduces sepsis-induced mortality in surgical patients (Bernard *et al*, 2001; Vincent *et al*, 2005).

The model of curative cancer treatment for locally advanced gastrointestinal cancer is increasingly a multimodal approach, with neoadjuvant chemotherapy, radiation therapy or a combination of both before resectional surgery. The treatment model for oesophageal cancer is ideal for the study of coagulation and VTE risk, for several reasons. The patient population is homogeneous, with an average age above 60 years and a duration of surgery between 4 and 7 h. The magnitude of surgical trauma is relatively uniform with respect to altered physiology, immune function and metabolism, with a marked pro-inflammatory cytokine response and a well-defined high risk of clinical complications (Enzinger and Mayer, 2003). Finally, neoadjuvant chemotherapy and radiation therapy may be associated with increased operative risks, and whether this relates to an altered pro-coagulant or immune response is unknown (Bosset *et al*, 1997; Reynolds *et al*, 2007).

There are no reported longitudinal studies of pro-coagulant response, immune response or clinical and subclinical VTE risks in multimodality treatment regimens, and no study in cancer surgical patients has evaluated these parameters beyond a month postoperatively. We report herein that neoadjuvant chemoradiation activates the pro-coagulant response, and that oesophageal cancer surgery is associated with a sustained activation of the response for up to 6 months postoperatively. All clinical VTE events occurred beyond the period of post-operative hospitalisation, and therefore these data may have implications for the duration of thromboprophylaxis.

## Patients and methods

### Patients

All patients with localised disease during the period from July 2006 to June 2007 were included in the study. Patients with biopsy-proven cancer of the oesophagus or an oesophago-gastric junction with localised disease determined by computerised tomography of the neck, thorax and abdomen, and whole-body ^18^FDG-PET scans, and considered resectable by the primary surgeon, were eligible. The inclusion criteria for multimodal therapy, approved by the institutional review board, are as follows: age <77 years; satisfactory performance status (ASA 1 or 2; Karnofsky >90%) and medical fitness for surgery; no previous chemotherapy or radiation therapy; a leukocyte count greater than 3500 per cubic millimetre, a platelet count above 100 000 per cubic millimetre; and serum creatinine less than 124 *μ*mol per litre (Reynolds *et al*, 2007).

The multimodality regimen was as previously described, with radiation (40–44 Gy per 15–22 fractions) over 3 weeks, and concurrent chemotherapy with 5-fluorouracil (15 mg kg^−1^) on days 1–5 and with cisplatin (75 mg m^−2^) on day 7; chemotherapy was repeated on week 6 (Reynolds *et al*, 2007). All patients underwent a thoracotomy as a part of their surgical management, either combined with an abdominal and neck exploration (three-stage) for mid- and upper-oesophageal cancers, or for cancer arising in the long-segment Barrett's oesophagus, or with an abdominal exploration (two-stage) for most lower third and junctional tumours, or combined with a total gastrectomy for junctional tumours with significant gastric extension. A two-field lymphadenectomy (abdominal and thoracic) was performed in all cases.

All patients underwent VTE prophylaxis with enoxaparin sodium (Clexane) 40 mg s.c. daily (sanofi aventis, Dublin, Ireland) only during hospitalisation for surgery. This was commenced at least 24 h before surgery and continued until discharge. All patients were fed through a needle-catheter jejunostomy, beginning on the first post-operative day, and continuing for a minimum of 10 days.

### Study design

Even in patients with adequate prophylaxis, studies suggest that outpatient VTE is more common than inpatient VTE after surgery, thereby challenging existing standards in VTE prophylaxis. We hypothesise that this is because of prolonged activation of the pro-coagulant response. The aim of this study was to prospectively examine the pro-coagulant and inflammatory response up to 9 months postoperatively in a cohort of patients treated with a multimodal regimen and curative intent for oesophageal cancer. The institutional review board approved this study. Thrombophilia testing (anti-thrombin activity, protein C activity, free and total protein S antigen, APC resistance, factor V Leiden mutation, Prothrombin 20210 mutation, anti-phospholipid antibody screen) was performed in all patients. In addition, each patient had the following studies conducted at baseline, after 1 week of chemoradiation and after the second cycle of chemotherapy, on pre-operative day 1 and postoperatively on days 1, 3, 7, 14, 21, 28, and at 2-, 3-, 6- and 9-month' follow-up: pro-thrombin time (PT), activated partial thromboplastin time (APTT), fibrinogen, FVIII, anti-thrombin, protein C, protein S, D-dimer, TAT complexes and pro-thrombin fragment 1+2 (F1+2) levels, a panel of cytokines and growth factors. NF*κ*B activation in peripheral blood mononuclear cells (PBMCs) was measured up to 2 months postoperatively.

### Pro-coagulant studies

Blood was added to tubes containing 0.109 M trisodium citrate anti-coagulant at a ratio of 9 : 1, centrifuged (4082 r.p.m.) for 20 min at 4°C, transferred to a pre-chilled tube and stored at −80°C until analysis. Automated functional assays for the quantitative determination of PT, APTT, protein C and protein S levels were performed using ACL 9000 Advance Instrumentation (Beckman Coulter, Dublin, Ireland). FVIII:C was measured by the one-stage clotting method, using FVIII-deficient substrate (Immuno, Vienna, Austria), as previously described (O'Donnell *et al*, 2001). F1+2 and TAT complexes were measured using commercially available ELISA kits (Dade Behring, Marrburg, Germany). Anti-thrombin antigen levels were determined using NOR Partigen Immunodiffusion Plates (Dade Behring).

### PBMC NF*κ*B activation

Lymphocytes were isolated using Lymphoprep solution (Axis-Shield, Oslo, Norway). Nuclear and cytoplasmic fractions were separated, and NF*κ*B and I*κ*B*α* were measured using the TransAM kit (Active Motif, Rixensart, Belgium). This assay contains an immobilised consensus binding site oligonucleotide to which the activated nuclear extract binds. The positive control used for NF*κ*B was Jurkat (TPA+CL) nuclear extract. Wild-type and mutated NF*κ*B consensus oligonucleotides were used to assess the oligonucleotide-binding specificity of the assay. Addition of primary antibody specific for either the NF*κ*B p65 or p50 subunit preceded a series of incubations and washes. A secondary antibody conjugated to horseradish peroxidase was then added to each well, subsequently covered and incubated for 1 h at room temperature without agitation, and washed four times with 200 *μ*l 1 × washing buffer. Finally, developing solution provided a sensitive chemiluminescent readout and absorbance was read on the spectrophotometer at 450 nm with a reference wavelength of 655 nm. TransAM Function ELISA (Active Motif, Carlsbad, CA, USA) was also used for the detection and analysis of I*κ*B*α* phosphorylation in cytoplasmic fractions.

### Cytokine analysis

Randox Evidence Investigator Biochip (Randox, Belfast, UK) array technology was used to assay cytokines. This allowed the simultaneous detection of multiple related cytokine and growth factors on the basis of immunoassays on a single sample. The Biochip used is a device containing an array of discrete test regions of immobilised antibodies specific to different cytokines and growth factors. A sandwich chemiluminescent immunoassay was used for the cytokine array. The cytokine array was quantitatively tested for each of the following growth factors and cytokines: IL-1a, IL-1*β*, IL-2, IL-4, IL-6, IL-8, IL-10, IL-12, IFN-*γ*, TNF-*α* vascular endothelial growth factor (VEGF) and epidermal growth factor.

### Clinical and radiological assessment

CT pulmonary angiography and venogram (CTPAV) was incorporated into the staging workup protocol performed on all patients with oesophageal cancer at the onset of the study. A post-operative CTPAV was carried out at 10 days in all recruited patients to assess for asymptomatic thrombus or for the presence of pulmonary microemboli. Further imaging in search of VTE was performed as clinically indicated.

All complications from surgery to discharge from hospital were prospectively documented. Respiratory failure was defined as the requirement for mechanical ventilation beyond 24 h after surgery. Acute respiratory distress syndrome and multiple organ failure were as defined by Bone *et al* (1992). Sepsis required evidence of SIRS with microbiological evidence of infection, and the diagnosis of pneumonia required either positive sputum cultures or a clear clinical and radiographic evidence of consolidation.

### Statistical analysis

Statistical analysis was carried out using SPSS version 12 for Windows (SPSS Inc., Chicago, IL, USA). Primarily, data were tested for normality. Many of the variables were found to have log-normal distributions. Therefore, these were transformed using a log transformation (log_10_) to run parametric tests on data. For parametric continuous variables, multiple repeated-measures analysis of variance (ANOVA) was used to compare the values preoperatively, with data measured at multiple subsequent time points. The Friedman test was used to examine changes over time for data that were not normally distributed. Categorical variables were compared using cross-tab analysis, and Pearson's *χ*^2^-tests were used to test for significance. Post-operative values were compared with those on pre-operative day 1. Values are mean±s.e.m. Significance was set at *P*<0.05.

## Results

### Patient demographics, surgical data and in-hospital complications

During a 12-month period (July 2006 to June 2007), 36 consecutive patients with localised oesophageal carcinoma who came to undergo surgery were enrolled in the study. The mean age was 62 years (range 35–77 years), with 24 men and 12 women. Among them, –22 patients had adenocarcinoma and 14 had squamous cell cancer. There was no in-hospital post-operative mortality, 3 patients (8%) had sepsis and 10 (26%) developed pneumonia. The median post-operative length of stay was 17 days (range, 14–34 days).

### Venous thromboembolism

All patients had negative initial screening for thrombophilia. No VTE was evident following induction chemoradiation before surgery, or during post-operative hospitalisation. CT pulmonary angiography and venogram on day 10 postoperatively identified no subclinical VTE. Four patients (11%) experienced non-fatal clinical VTE after discharge from hospital. These events were a proximal DVT (59-year-old man, day 57 postoperatively), bilateral pulmonary emboli (52-year-old man, day 45 postoperatively), a right calf DVT and bilateral pulmonary emboli (37-year-old man, day 43 postoperatively), and a proximal DVT and unilateral pulmonary embolus (54-year-old man, day 68 postoperatively).

### Neoadjuvant chemoradiation before surgery

Compared with baseline, both the first cycle of combination chemoradiation and the second cycle of chemotherapy resulted in significant (*P*<0.05) increases in FVIII:C, D-dimers, TAT, F1+2 and NF*κ*B activation (p50) (Table 1). In contrast, significant (*P*<0.05) reductions were observed in PT, APTT and protein S levels. For cytokines and growth factors, there was a significant (*P*<0.05) increase in IL-8 after the first cycle of CRT only, and a significant increase in IL-6 after both cycles. There was a significant reduction in VEGF after induction chemoradiation and after the second cycle of chemotherapy. All parameters had returned to values not significantly above baseline when tested the day before surgery.

### Post-operative responses

#### Pro-coagulant response

**Figure 1:**
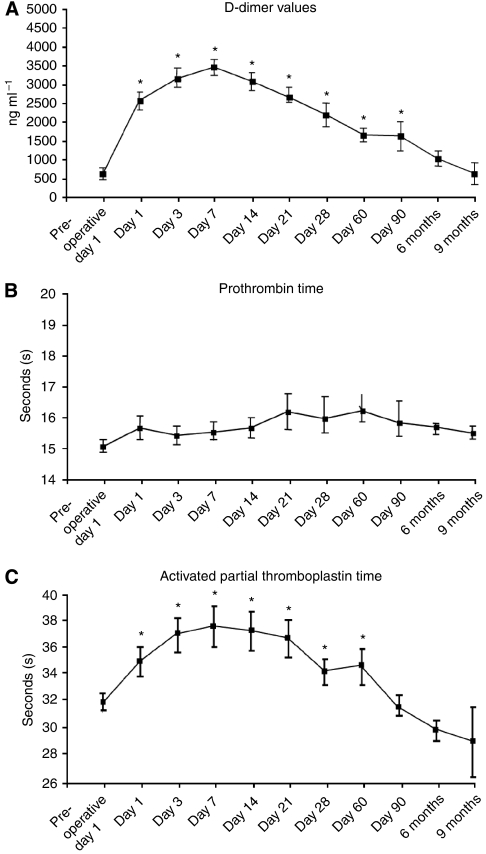
(**A**) D-Dimers were significantly elevated (^*^P<0.0001) in the early post-operative period (days 1–7), compared with pre-operative baseline, and remained elevated up to 3 months postoperatively before returning to baseline (normal range, 0–300 ng ml^−1^). (**B**) No significant difference in pro-thrombin time was observed at any post-operative time point (*P*=NS) (normal range, 12–15 s). (**C**) Activated partial thromboplastin time (APTT) was significantly (^*^P<0.05) elevated postoperatively and remained significantly elevated up to post-operative day 60 (normal range, 25–35 s).

**Figure 2:**
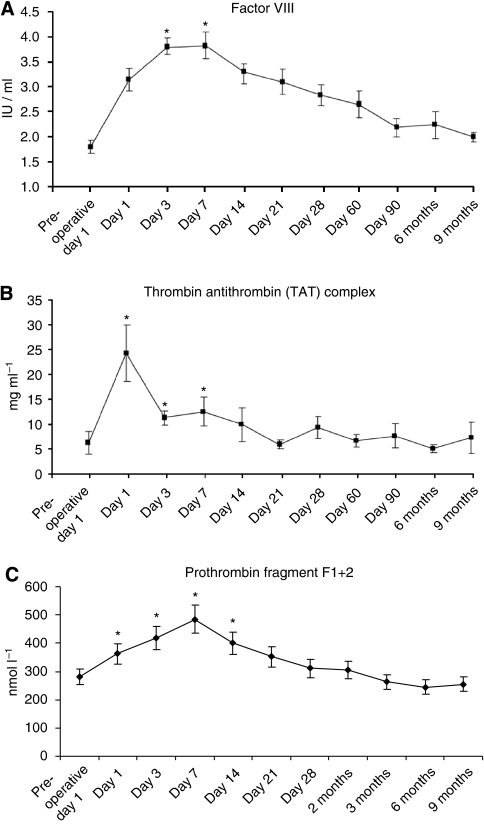
(**A**) A significant elevation in Factor VIII:C was observed in the early post-operative period (^*^P<0.05), and this level remained elevated up to 6 months postoperatively (normal range, 0.55–2.05 IU ml^−1^). (**B**) The thrombin-anti-thrombin (TAT) complex was significantly increased in the early post-operative period (^*^P<0.05 up to day 7) but returned to baseline levels thereafter (normal range, 1–4.1 mg ml^−1^). (**C**) Pro-thrombin factor F1+2 was also significantly elevated postoperatively compared with pre-operative baseline (^*^P<0.05), and remained significantly elevated up to day 14 postoperatively (normal range, 0.24–1.44 nmol l^−1^).

D-dimers were significantly (*P*<0.0001) increased compared with pre-operative levels at all time points up to 3 months postoperatively (Figure 1A). There was no significant change in PT at any time point (Figure 1B); however, APTT was significantly (*P*<0.05) increased up to day 60 postoperatively (Figure 1C), returning to baselines levels thereafter. Factor VIII (VIII:C) was significantly (*P*<0.05) increased up to 6 months postoperatively (Figure 2A). The thrombin-anti-thrombin complex (Figure 2B) and F1+2 (Figure 2C) were significantly (*P*<0.05) increased up to days 7 and 21, respectively.

#### Anti-coagulant response

**Figure 3:**
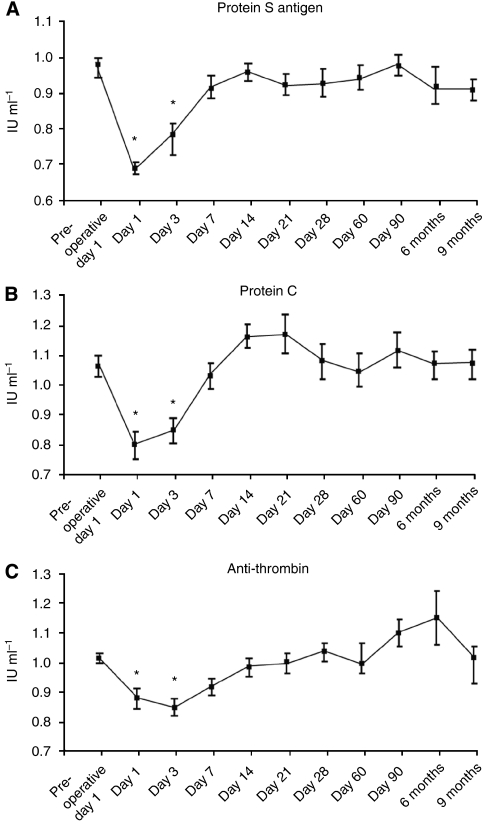
The levels of the natural anti-coagulants, anti-thrombin (normal range, 0.83–1.15 IU ml^−1^) (**A**), protein C (normal range, 0.74–1.32 IU ml^−1^) (**B**) and protein S (normal range, 0.74–1.32 IU ml^−1^) (**C**) were all significantly (^*^P<0.05) decreased in the early post-operative period (days 1–7) but returned to normal levels after post-operative day 7.

A significant (*P*<0.05) decrease in plasma anti-thrombin, protein C and protein S levels was observed on days 1 and 3 postoperatively, compared with pre-operative day 1 (Figure 3). However, these levels had all returned to baseline values by day 14.

#### Immunoinflammatory response

**Figure 4:**
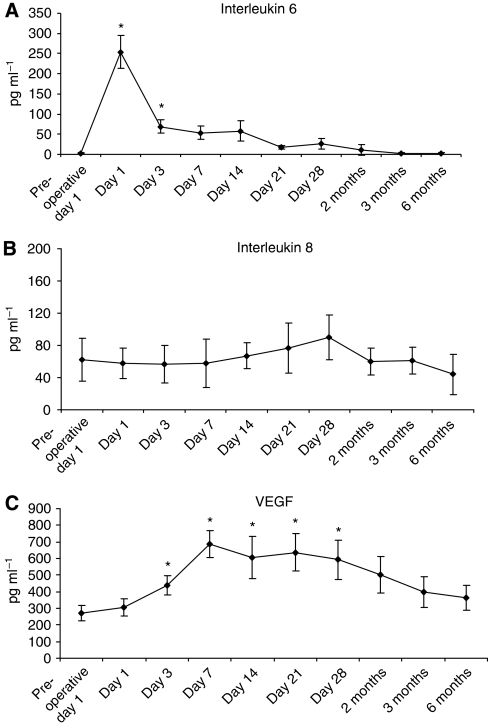
Cytokine analysis was performed using the Randox Evidence Investigator biochip arrays. (**A**) IL-6 levels were significantly (^*^P<0.05) increased in the immediate post-operative period (days 1 and 3), but the levels returned to normal by post-operative day 7; (**B**) there was no significant change in post-operative IL-8 expression (*P*=NS); (**C**) VEGF levels were significantly (*P*<0.05) elevated postoperatively up to post-operative day 28, after which the levels declined.

**Figure 5:**
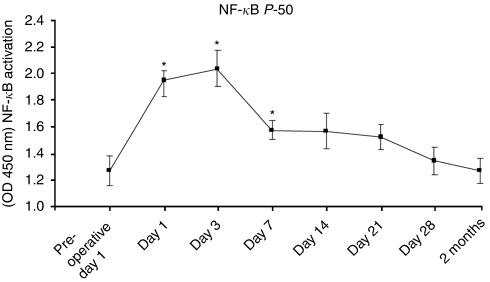
NF*κ*B activation was assessed using the TransAm assay. Surgery resulted in a significant elevation in NF*κ*B activation (^*^P<0.05) in the early post-operative period (days 1 and 3), which returned to baseline thereafter.

Of the 14 cytokines and growth factors measured, IL- 6, IL-8 and VEGF showed consistent and reliable measurements in this model. Following oesophagectomy, there were significant (*P*<0.05) elevations of IL-6 from baseline pre-operative levels noted in both cohorts on day 1 and the increase in IL-6 was sustained up until 1 month post-operatively (Figure 4A). Levels of IL-8 were not significantly altered postoperatively (Figure 4B). Vascular endothelial growth factor levels were significantly (*P*<0.05) increased in both cohorts up to 3 months postoperatively, and there was no difference between the two cohorts (Figure 4C). Activation of NF*κ*B p50 was manifest in the post-operative period, with significant (*P*<0.0001) increases from baseline levels recorded throughout the first week (Figure 5).

## Discussion

There is an intriguing association between cancer and VTE, with increased risks of VTE in patients with cancer, a high prevalence of occult cancer in patients presenting with idiopathic VTE, and some evidence that anti-coagulation can affect cancer biology and improve outcomes (Kakkar *et al*, 2004; Lee *et al*, 2005; Behanwala and Williamson, 2009). Although Virchow and Trousseau, respectively, provided insights into the pathophysiology of VTE and its association with cancer in the mid-nineteenth century, significant gaps exist in the understanding of cancer-associated VTE. Most scientific research is limited to studies on patients with advanced malignancies, rather than on patients undergoing curative approaches, and there is little scientific and clinical understanding of the impact of multimodal curative approaches. Moreover, there is emerging consensus that the lack of application of appropriate thromboprophylaxis in cancer patients may underlie significant morbidity and mortality. The ENDORSE (Epidemiological International Day for the Evaluation of Patients at Risk for VTE in the Acute Hospital Care Setting) study included 19 842 surgical patients, of whom only 11 613 (58.5%) received American College of Chest Physicians (ACCP)-recommended thromboprophylaxis (Cohen *et al*, 2008). Recent studies suggest that, even in patients with adequate prophylaxis, outpatient VTE is more common than inpatient VTE after surgery, thereby challenging the existing standards in VTE prophylaxis (Spencer *et al*, 2007). To the best of our knowledge, this is the first study to systematically follow through a cohort of patients treated with a multimodal regimen and curative intent. The finding of an activated pro-coagulant response after induction chemoradiation and a sustained response for up to 6 months postoperatively suggests that trials evaluating extended VTE prophylaxis beyond current standards should be considered.

Oesophagectomy is an exemplar model of complex major surgery; there is no common elective oncological resection that carries the same operative risks, with a 10 and 14% in-hospital mortality rate reported from recent North American and UK series, respectively (Bailey *et al*, 2003; Enzinger and Mayer, 2003; McCulloch *et al*, 2003). After the period of post-operative hospitalisation, a further 20–40% of patients treated with curative intent die within 1 year (Enzinger and Mayer, 2003). In many cases, cancer recurrence will clearly be the cause of death, but VTE, both evident and silent, may be causative, particularly in cases in which death occurs in the absence of obvious disease recurrence. In this study, the incidence of symptomatic VTE was 11%. Screening for subclinical VTE was not continued after post-operative day 10 within the design of this study. Inherited thrombophilia was excluded in all patients. In this study, all patients received post-operative VTE prophylaxis with high-dose low-molecular-weight heparin (LMWH), and this was continued for a median of 17 days with a range of 14–34 days. The median duration of thrombophylaxis in the majority of patients was shorter than the 28 days supported by the ENOXACAN II and FAME trials (Bergqvist *et al*, 2002; Rasmussen *et al*, 2006). These positive studies compared short-course with 1-month VTE prophylaxis and evaluated clinical and subclinical VTE events in the first month postoperatively, and the ENOXACAN II study revealed an approximate 60% reduction in VTE events with the longer prophylaxis that persisted at 3 months of follow-up (Bergqvist *et al*, 2002). In this study, no clinical or silent VTE was evident by CTPAV on postoperative day 10. It is noteworthy that all clinical VTE events occurred in the second and third months postoperatively. In the laboratory analysis, the early inhibition of the anti-coagulant response, and some elements of an activated pro-coagulant response, including F1+2 and TAT, returned to normal before discharge. Therefore, these increases may be an acute response to the surgical insult, and it is possible that they are not related to long-term pro-coagulant activation in this cohort. However, D-dimers and FVIII:C, for 3 and 6 months, respectively, persisted during a period when patients were not receiving VTE prophylaxis, and during which the clinical events occurred. The proof of concept that this pro-coagulant profile translates to a risk of VTE in patients after oesophageal or other complex cancer surgery requires further study; nonetheless, we can speculate that this inference is reasonable as FVIII:C is associated with an increased risk of VTE. In a study of 19 237 adults with no baseline history of VTE, cancer or warfarin use, 159 VET events occurred and elevated FVIII:C levels were common, independent and dose-dependent risk factors for VTE (Tsai *et al*, 2002).

The clinical relevance of a sustained post-operative pro-coagulant response is that recent evidence indicates that outpatient VTE is three-fold more common than inpatient VTE, and approximately half of the outpatients with VTE were recently hospitalised (Spencer *et al*, 2007). In this study, all VTE episodes were in the outpatient setting, in patients who were not on LMWH prophylaxis. As the majority of VTE events occur in the outpatient setting and the pro-coagulant markers remained elevated for up to 6 months in this study, extension of prophylaxis after discharge may be warranted in this and other high-risk models. However, the logistics of such studies are daunting, as a randomised trial comparing 1 month with 3 months of VTE prophylaxis, with significant power to reveal a 20% reduction in clinical VTE events (from 11 to 8.8%), would require 3000 patients per treatment arm. Studies powered to include subclinical events and surrogate pro-coagulant markers may have acceptable rationale and less logistical difficulties.

The design of the study allowed us to address the impact of neoadjuvant chemoradiation on pro-coagulant response before surgical resection. Radiotherapy and chemotherapy may contribute to pro-thrombotic risk (Kuzel *et al*, 1990; Holm *et al*, 1996; Tufia *et al*, 2006). Chemotherapy, including drugs cisplatin and fluorouracil used in the multimodality regimen, can induce thrombogenic effects through multiple mechanisms, including tumour cell secretion of immunomodulatory and pro-angiogenic cytokines, increased tissue factor expression on vascular endothelial cells, direct vascular endothelial toxicity and reduced protein C (Kuzel *et al*, 1990; Tufia *et al*, 2006) In this study, an upregulated pro-coagulant response was evident even after 1 week of combination chemoradiation, associated with elevated FVIII:C, F1+2 and TAT levels, with a corresponding reduction in protein C. These patients did not receive VTE thromboprophylaxis, and no clinical episodes of VTE were recorded. Hence, the clinical relevance before surgery is unclear. In addition, whether this pro-coagulant effect translates into a heightened post-operative response compared with a group undergoing surgery alone may merit study. Recent reports indicate that VTE may occur after radical chemoradiation in patients with advanced oesophageal cancer, and that this is associated with decreased overall survival (Tetzlaff *et al*, 2007, 2008).

A cross talk between inflammation and coagulation was suggested by the findings of this study. Cytokines associated with inflammation, in particular TNF-*α* and IL-1, have been shown to decrease plasma levels of critical anti-coagulant proteins, including anti-thrombin and protein C, and to increase tissue factor expression (Esmon, 2005). Furthermore, APC, which can reverse these effects in sepsis, works in part through the inhibition of NF*κ*B activation in inflammatory cells. In this study, the increase in NF*κ*B and decrease in protein C were matched in the early response to surgery.

In conclusion, this prospective longitudinal study showed that induction chemoradiation induced a pro-coagulant and inflammatory response, and that sustained pro-coagulation was evident for 3–6 months postoperatively. The entire period of the multimodal regimen, and perhaps up to 6 months afterwards, may represent a high-risk period for VTE. Scientific and clinical studies in this model and in other long and complex multimodal regimens merit further investigation, in particular to address the duration of appropriate thromboprophylaxis. The relatively low rate of clinical VTE in this study suggests that subclinical events and surrogate markers should be included in the design of such studies.

## Figures and Tables

**Table 1:** Neoadjuvant chemoradiation before surgery

**Procoagulant**	**Baseline**	**First cycle**	**Second cycle**
D-Dimers (ng ml^−1^)	629±59.02	748±48.35^a^	1023±129.68^a^
Pro-thrombin time (s)	15±1.08	14±0.77	13±1.05
APTT (s)	32±1.02	30±0.83^a^	29±0.91^a^
Fibrinogen (g l^−1^)	4.19±0.67	4.59±0.20	4.35±0.19
Factor VIII (IU ml^−1^)	1.80±0.11	2.29±0.21^a^	2.71±0.30^a^
Thrombin-anti-thrombin (TATC) (*μ*g ml^−1^)	6.22±0.05	7.61±0.05^a^	8.21±0.06^a^
Pro-thrombin fragments 1 and 2 (IU ml^−1^)	285.29±3.6	297.46±2.8^a^	347.62±11.6^a^
			
*Anticoagulant*			
Protein C (IU ml^−1^)	1.06±0.05	1.01±0.05	1.05±0.06
Protein S (IU ml^−1^)	0.98±0.06	0.88±0.03^a^	0.90±0.05^a^
Anti-thrombin (IU ml^−1^)	1.02±0.03	1.03±0.03	1.05±0.04
Platelets (10^9^ l^−1^)	267±23.6	207±16.8^a^	219±34.9
			
*Cytokines and growth factors*			
IL-6 (pg ml^−1^)	4.13±1.08	7.60±1.07^a^	9.61±1.25^a^
IL-8 (pg ml^−1^)	70.82±34.95	117±5.21^a^	71.24±15.35
IL-10 (pg ml^−1^)	1.09±0.22	0.64±0.19	0.83±0.17
VEGF (pg ml^−1^)	381±67.4	295±65.4^a^	286±60.9^a^
			
NF*κ*B activation (p50)(OD450 nm)	1.27±0.13	1.71±0.17^a^	1.75±0.12^a^

Abbreviation: APTT=activated partial thromboplastin time.

a *P*<0.05 compared with baseline.
